# Was It an Adrenocortical Adenoma or an Adrenocortical Carcinoma? Limitation of the Weiss Scoring System in Determining the Malignant Potential of Adrenocortical Tumor: Report on Two Cases

**DOI:** 10.1155/2022/7395050

**Published:** 2022-09-14

**Authors:** Cheuk-Lik Wong, Chun-Kit Fok, Yuk-Kit Chan, Vicki Ho-Kee Tam, Lai-Ming Fung

**Affiliations:** ^1^Department of Medicine and Geriatrics, Caritas Medical Centre, Shamshuipo, Kowloon, Hong Kong; ^2^Department of Medicine and Geriatrics, Pok Oi Hospital, Yuen Long, The New Territories, Hong Kong

## Abstract

**Background:**

Adrenocortical carcinoma (ACC) is a rare endocrine malignancy. An accurate diagnosis of ACC is of paramount importance as it greatly impacts the management and prognosis of a patient. However, the differentiation between early stage, low-grade ACC and adrenocortical adenoma (ACA) may not always be straightforward. The recommended classification system, namely, the Weiss scoring system, is not without flaws. We herein report two cases of ACC which were initially diagnosed as ACA according to the Weiss scoring system but developed distant metastases in subsequent years. *Case Presentation*. Case 1: A 60-year-old Chinese woman presented with a recent onset of worsening of blood pressure control and clinical features of Cushing's syndrome. Investigations confirmed ACTH-independent endogenous hypercortisolism, and a CT abdomen showed a 6 cm right adrenal mass. Twenty-four-hour urine steroid profiling revealed co-secretion of adrenal androgens and atypical steroid metabolites. Laparoscopic right adrenalectomy was performed, and pathology of the tumor was classified as an ACA by the Weiss scoring system. Four years later, the patient presented with an abrupt onset of severe hypercortisolism and was found to have a metastatic recurrence in the liver and peritoneum. The patient received a combination of mitotane, systemic chemotherapy, and palliative debulking surgery and succumbed 8.5 years after the initial presentation due to respiratory failure with extensive pulmonary metastases. *Case 2*: A 68-year-old Chinese woman presented with acute bilateral pulmonary embolism and was found to have a 3 cm left adrenal mass. Hormonal workup confirmed ACTH-independent endogenous hypercortisolism, and laparoscopic left adrenalectomy revealed an ACA according to the Weiss scoring system. Five years later, she presented with recurrent hypercortisolism due to hepatic and peritoneal metastases. The patient had progressive disease despite mitotane therapy and succumbed 7 years after initial presentation.

**Conclusions:**

Although the Weiss scoring system is recommended as the reference pathological classification system to diagnose adrenocortical carcinoma, there remain tumors of borderline malignant potential which may escape accurate classification. Various alternative classification systems and algorithms exist but none are proven to be perfect. Clinicians should recognize the potential limitation of these histological criteria and scoring systems and incorporate other clinical parameters, such as the pattern of hormonal secretion, urinary steroid profiling, and radiographic features, to improve the prognostication and surveillance strategy of these tumors.

## 1. Background

Adrenocortical carcinoma (ACC) is a rare, highly aggressive malignancy with a reported annual incidence of 0.7–2 per one million people, while adrenocortical adenoma (ACA) is one of the commonest adrenal tumors and is benign [1, 2]. An accurate diagnosis of ACC is of paramount importance as it greatly impacts the management, prognosis, and surveillance strategy of a patient. Although ACC can be readily diagnosed in the presence of metastases, the differentiation between early stage ACC and ACA could be challenging histologically, especially in the setting of a well-differentiated low-grade ACC [3–5]. The Weiss scoring system, first proposed in 1984 and subsequently modified in 2002, is the most widely employed pathology classification system in adrenocortical tumors and is recommended as the gold standard for the diagnosis of ACC by international guidelines [2, 6, 7]. Nine histological criteria, which are based on the morphological assessment of tumor structure, cytological features, and invasive tumor properties under light microscopy, are graded by the pathologist, and a diagnosis of malignancy is made if three or more criteria are present [6]. Despite a very high diagnostic performance, 100% sensitivity and specificity could not be achieved. Factors affecting its diagnostic performance include tumors of borderline malignant potential with only one or two fitting criteria, interobserver reproducibility, adequacy of tumor sampling, low applicability among nonexpert pathologists, and ACC variants [8–11]. Other histopathological algorithm and scoring system, namely, the reticulin algorithm and Helsinki score, are recently developed and validated [12, 13]. They are shown to be highly accurate in differentiating between benign and malignant adrenocortical tumors with almost 100% or nearly 100% sensitivity and specificity, but their routine use is yet to be widely adopted [14]. Nevertheless, certain clinical, biochemical, and radiological features may provide physicians with important complementary information to predict the malignant potential of an adrenocortical tumor. We therein report two cases with presumable ACA years ago and several years later presented with Cushing's syndrome and metastatic ACC, highlighting the limitation of the Weiss scoring system and the importance of integrating clinical, biochemical, radiological, and histological information to accurately determine the malignant potential of an adrenocortical tumor.

## 2. Case Presentation

### 2.1. Case 1

**Figure 1:**
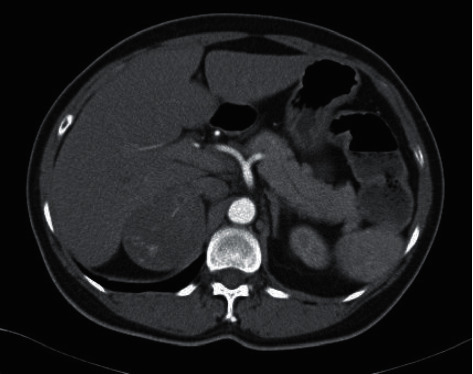
CT abdomen of Patient A on 9 August 2012. A 6.4 × 5.2 × 5.8 cm heterogeneously enhancing mass in the right adrenal gland with pre-contrast HU 35.4 (red arrow).

Patient A, a 60-year-old Chinese lady, who had a history of gastroesophageal reflux disease, hypertension, and hyperlipidemia, presented with insidious onset of right loin pain for 3 months in June 2012. She also had worsening of blood pressure control over the past few months and occasional palpitations. Physical examination was notable for moon face and buffalo hump suggesting Cushing's syndrome. Computed tomography (CT) of the abdomen performed for suspected urinary tract pathology showed a 6 cm right adrenal mass (Figure 1). Subsequent hormonal investigations (Table 1) confirmed the diagnosis of ACTH-independent Cushing's syndrome, which was likely due to a cortisol-secreting adrenal tumor. There was also co-secretion of adrenal androgens and atypical steroid metabolites.

The patient underwent laparoscopic right adrenalectomy in August 2012. The pathology of the resected tumor was reported to be ACA. Postoperatively, hypertension was cured and antihypertensives were stopped. A follow-up CT at 9 months after the operation did not show any evidence of recurrence, and urine steroid profiling (USP) at 1.5 years postoperatively showed normalization of the previously abnormal steroid metabolites together with normal free cortisol and androgens. She also developed adrenal insufficiency and was maintained on hydrocortisone replacement postoperatively until 2015.

**Figure 2:**
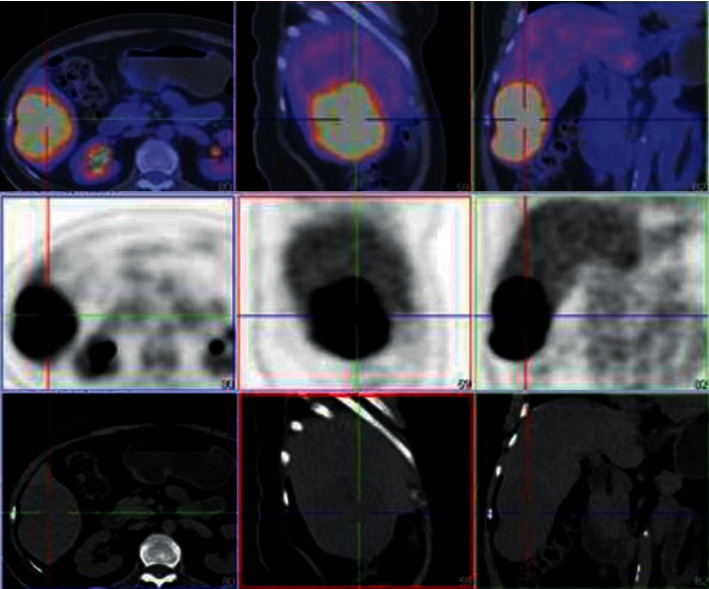
^18^FDG-PET-CT scan of Patient A on 6 July 2016. CT abdomen of Madam F on 9 August 2012. An 8 cm liver mass (SUVmax 8.4) was found at segment 8.

**Figure 3:**
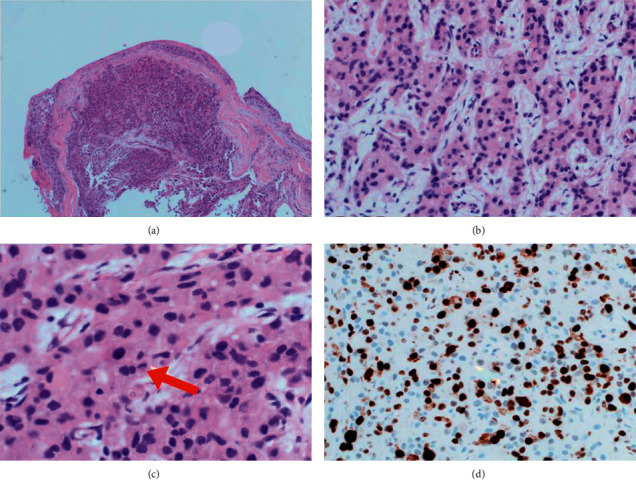
Photomicrographs of peritoneal nodule biopsy of Patient A. (a) Peritoneal nodule section (H&E × 40). (b) Tumor cells arranged in the cords and trabeculae (H&E × 200). (c) Hyperchromatic nuclei of tumor cells. The presence of mitosis was noted in a tumor cell (red arrow) (H&E × 400). (d) Ki-67 proliferative index was 50% (IHC × 400).

In May 2016, the patient presented to us with a relatively acute onset of ACTH-independent hypercortisolism with marked hypertension and fluid overload in addition to clinical (hirsutism) and biochemical hyperandrogenism (Table 1). ^18^F-fluorodeoxyglucose positron emission tomography/computed tomography (^18^FDG-PET/CT) scan showed a large solid mass at the right lobe of the liver (maximum standardized uptake value [SUV_max]_ 8.4), a small nodule around the previous right adrenalectomy bed (SUV_max_ 2.1), and multiple foci of hypermetabolic peritoneal lesions in both sides of the abdominal cavity (highest SUV_max_ 4.6) (Figure 2). A laparoscopic peritoneal biopsy was performed. Histologically, the biopsy showed a metastatic carcinoma composed of large polygonal cells with abundant pink cytoplasm arranged in trabeculae infiltrating a fibromyxoid stroma. Nuclear hyperchromatism and prominent nucleoli were evident in the carcinoma cells. Immunohistochemical studies showed positivity towards synaptophysin, Melan A, and inhibin. Ki-67 proliferative index was 50% (Figure 3). A pathological diagnosis of metastatic adrenocortical carcinoma was made.

**Figure 4:**
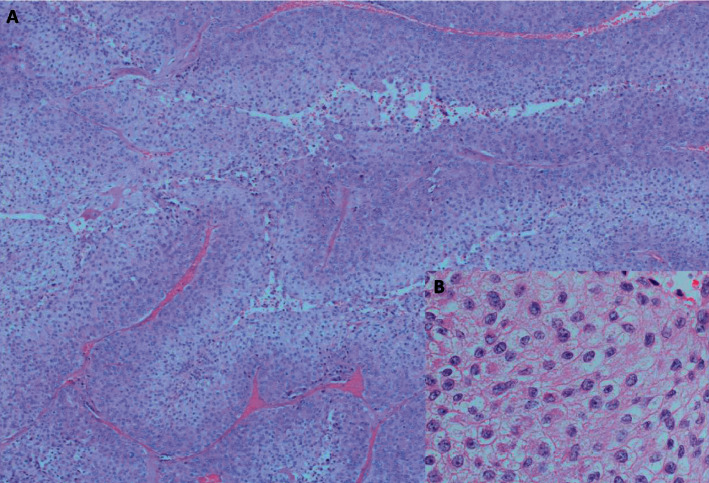
Photomicrographs of right adrenal tumor of Patient A on pathology review. (a) Diffuse growth pattern (H&E × 40). (b) Regular nuclei and eosinophilic cytoplasm of tumor cells (H&E × 400).

We retrospectively reviewed the histology of the right adrenal tumor which was removed in August 2012 and compared it with that of the peritoneal nodule. The morphological appearance of the previous adrenal tumor shared a focal resemblance with that of the peritoneal nodule, suggesting that the current tumor might be arising from the original one, which would have been a well-differentiated ACC and had presently recurred and metastasized. The focal myxoid change was observed, and mitosis was up to 4/50 high power field (HPF). Atypical mitosis, necrosis, broad fibrous bands, vascular invasion, or capsular invasion was not identified. Although frankly malignant features were not present in the original adrenal tumor, the presence of a diffuse growth pattern and <25% clear tumor cells (Figure 4), which were not mentioned previously, were suspicious of malignancy.

Mitotane was initiated once the diagnosis was made in July 2016 with the dose titrated to maintain a target mitotane level of 14–20 micrograms/ml. A follow-up CT abdomen 4 months later showed progressive disease with enlarging liver metastasis (up to ∼10 cm) and peritoneal nodules. Chemotherapy with etoposide (EP) and cisplatin was added in January 2017, and follow-up CT 2 months later showed a reduction of peritoneal metastases and stable liver metastasis. EP was continued for five cycles until May 2017, but in July 2017, progressive disease was noted again on interval imaging with enlarging liver metastasis. At the same time, the patient became highly symptomatic with marked hypercortisolism which was uncontrolled despite high-dose mitotane (up to 4.5 gm/day) and metyrapone (up to 6 g/day).

A decision on palliative right hepatectomy and peritoneal nodule resection was made after multidisciplinary discussion and the operation was performed in September 2017. Further immunohistochemical studies showed intact immunoreactivity of mismatch repair proteins (MLH1, PMS2, MSH2, and MSH6) and less than 1% PD-L1 expression. Targeted next-generation sequencing on extracted tumor cell DNA did not reveal any variants or small insertion/deletion in the following genes: *AKT1, AKT3, ALK, ARAF, AURKA, BAP1, BRAF, BRCA1, BRCA2, CBFB, CCND1, CCND2, CCND3, CCNE1, CD79A, CD79B, CDH1, CDK4, CDK6, CDKN2A, CSF1R, CTNNB1, DDR2, EGFR, ERBB2, ERBB3, ERBB4, ESR1, EZH2, FGFR1, FGFR2, FGFR3, FLT1, FLT3, FLT4, GNA11, GNAQ, HGF, HIF1A, HRAS, IDH1, IDH2, IGF1R, JAK1, JAK2, JAK3, KDM6A, KDR, KIT, KRAS, LRRK2, MAP2K1, MAP2K2, MAP2K4, MAP3K1, MAP3K9, MAPK1, MAPK3, MET, MPL, MST1R, MTOR, MYD88, NF1, NOTCH1, NOTCH2, NOTCH3, NRAS, NRG1, NTRK1, NTRK2, NTRK3, PAK6, PDGFRA, PIK3CA, PIK3R1, PTCH1, PTEN, PTK2, PTK2B, RAF1, RET, RHOA, ROS1, SMO, SRC, STK11, SYK, TOP1, TSC1,* and *TSC2*. The postoperative course was complicated with bilateral subphrenic collection, left pleural effusion, pulmonary embolism, and steroid withdrawal syndrome which necessitated several drainage procedures, anticoagulation, and prolonged hospitalization for 3 months. After extended rehabilitation, the patient gradually regained independent self-care and functional ability.

In April 2018 (about 7 months after palliative debulking surgery), Patient A was found to have bilateral lung metastases. Chemotherapy was resumed with EP and subsequently the addition of doxorubicin (i.e., EDP) in view of progression in October 2018. She completed 6 cycles of EDP in May 2019 and enjoyed stable disease until August 2019, when she had disease progression with increasing pulmonary and peritoneal metastases. Gemcitabine and carboplatin were initiated as second-line palliative chemotherapy in September 2019. However, disease progression was again noted after 3 cycles, and chemotherapy was stopped. Patient A was then offered palliative care and she subsequently passed away due to respiratory failure with extensive pulmonary metastases in January 2021, almost 5 years after the relapse of the disease and 9 years after her initial presentation.

### 2.2. Case 2

**Figure 5:**
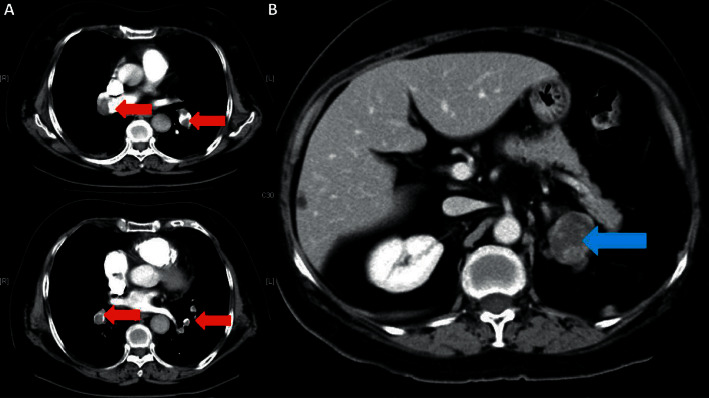
(a) CT pulmonary artery of Patient B on 25 August 2011 showing extensive bilateral pulmonary embolism (red arrow). (b) CT abdomen of Patient B on 13 October 2011. A 3.6 × 3.5 cm lobulated heterogeneously enhancing lesion with a focal calcified spot at the lateral limb of left adrenal with 30HU in pre-contrast scan (blue arrow).

Patient B, a 68-year-old Chinese lady, whose past medical history was remarkable for hypertension, presented to us in August 2011 for acute shortness of breath, chest discomfort, and transient loss of consciousness. She was later found to have extensive bilateral acute pulmonary embolism and was treated with intravenous thrombolysis followed by anticoagulation (Figure 5(a)). After stabilization, an abdominal US examination done for malignancy screening showed a 3 cm left suprarenal which was later confirmed to be a left adrenal mass on a CT scan (Figure 5(b)). Further examination of the patient showed clinical features of Cushing's syndrome which was confirmed with hormonal testing (Table 2). ACTH was suppressed, pointing to a diagnosis of a cortisol-producing adrenal tumor, and USP only showed elevated free cortisol and cortisol metabolites.

**Figure 6:**
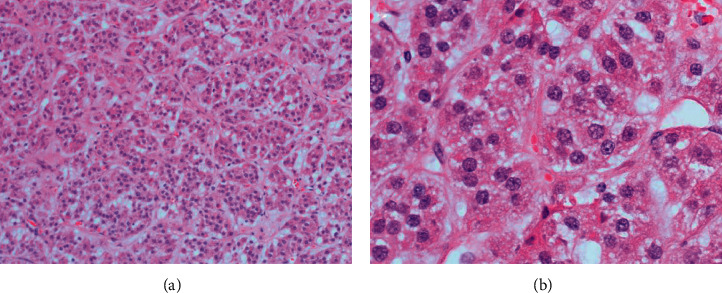
Photomicrograph of left adrenal tumor of Patient B. (a) Tumor cells in trabecular pattern (H&E × 100). (b) Tumor cells demonstrate nuclei of variable size and eosinophilic granular cytoplasm (H&E × 400).

Laparoscopic left adrenalectomy was performed in May 2012. Intraoperatively, a 5 cm left adrenal tumor was found and removed. Histologically, the lesion consisted of nests and trabeculae of medium-sized tumor cells and a rich capillary network. The tumor cells were polygonal, with pale to amphophilic cytoplasm, as well as round, deeply stained nuclei with nuclear pleomorphism (Figure 6). The maximal mitotic count was 5 in 50 HPFs. One fibrous septum thicker than 1 HPF was noted. There were also some areas of myxoid change and a large area of old hemorrhage while necrosis was not seen. A pathological diagnosis of an adrenocortical tumor was made, and the tumor was classified as an adenoma (ACA) according to the Weiss system. The patient was put on hydrocortisone 10 mg twice a day for postoperative adrenal insufficiency and morning cortisol remained low at 156 nmol/L in August 2016.

**Figure 7:**
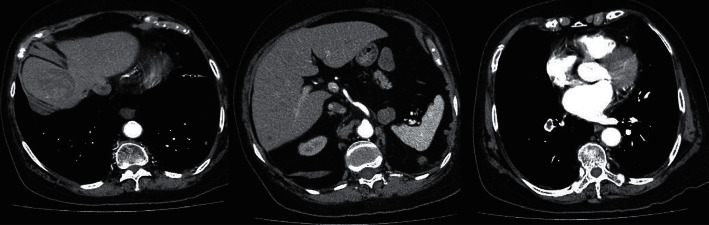
CT abdomen of Patient B on 30 December 2016. (a) Presence of liver metastasis. (b) Nodule at left adrenal bed and peritoneal nodule posterior to spleen. (c) Right descending pulmonary artery embolism.

**Figure 8:**
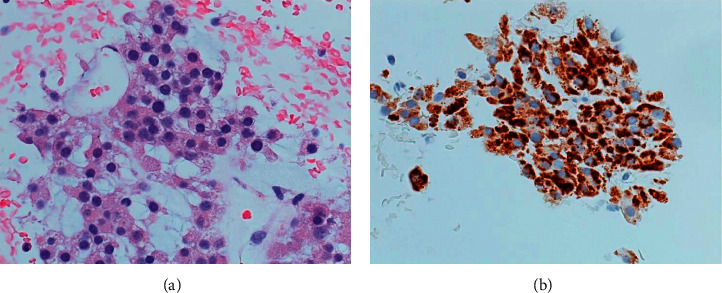
Photomicrograph of liver mass biopsy of Patient B. (a) Tumor cells with round nuclei and finely vacuolated cytoplasm that were arranged in anastomosing cords with delicate vasculature, resembling the previous left adrenal tumor (Figure 6) (H&E × 400). (b) Immunohistochemical staining showed diffuse positivity for Melan A.

However, in November 2016, the patient was noted to have the reappearance of Cushing's syndrome with central obesity and buffalo hump. Repeat hormonal testing showed relapse of endogenous ACTH-independent Cushing's syndrome as well as the presence of markedly elevated 17-hydroxyprogesterone (17-OHP) metabolites that were not seen in the previous urinary steroid profiling. An abdominal CT showed local recurrence of a 2 cm nodule at the left adrenal bed, a 4 cm liver mass, multiple peritoneal nodules, and recurrent pulmonary embolism (Figure 7). Apixaban was started for the treatment of pulmonary embolism, and US-guided fine needle aspiration of the liver mass in February 2017 showed tumor cells with round nuclei and finely vacuolated cytoplasm that were arranged in anastomosing cords with delicate vasculature, resembling the previous left adrenal tumor (Figure 8). Immunohistochemical studies revealed strong and diffuse staining for Melan A and weak staining for inhibin in the tumor cells, which were consistent with an adrenocortical origin, confirming the diagnosis of metastatic adrenocortical carcinoma.

**Figure 9:**
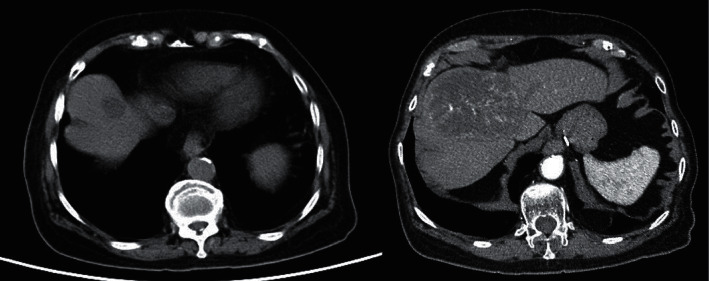
(a) Non-contrast CT abdomen of Patient B in October 2017 showing reduction of liver metastasis 8 months after mitotane (compared with Figure 7). (b) Contrast CT abdomen of Patient B in April 2018 showing the progression of liver metastasis 14 months after mitotane.

Patient B declined surgical debulking of the metastases due to old age (73 years old at that time) and risk of open surgery, while percutaneous radiofrequency ablation was deemed not feasible anatomically. Mitotane was thus started as monotherapy in February 2017 and titrated gradually to 3 g daily, which was the maximally tolerated dose to achieve a mitotane level of approximately 11 micrograms/ml. Follow-up ^18^FDG-PET/CT 8 months later (in October 2017) showed a mild interval reduction of liver metastasis (Figure 9(a)) together with stable disease in the left adrenal bed and peritoneal metastases. Despite the initial response, Patient B experienced significant progression of the disease since April 2018 with the growth of liver metastasis up to about 8 cm (Figure 9(b)). Systemic chemotherapy was also declined by the patient and she was managed medically with palliative intent. Patient B subsequently ran a downhill course and passed away in October 2018, 2 years after the recurrence of her disease and 7 years after her initial presentation.

## 3. Discussion

Histopathology remains the gold standard for diagnosing ACC and should be obtained in all patients [2]. Despite the fact that ACC often manifests clinical aggressiveness, its histological differentiation from benign ACA is not always straightforward, especially in a patient with early stage, low-grade disease which may behave more indolently [3, 15]. About 10% of ACC may be misdiagnosed according to two large observational cohorts. In the German ACC Registry, 21 of 161 patients (13%) had the diagnosis of ACC revised by the reference pathologist, while a misdiagnosis rate of 9% (26 out of 300 patients) was observed in another large Italian series [10, 16]. Several classification systems (Table 3) have been proposed for distinguishing between benign and malignant adrenocortical tumors, with varying sets of histological criteria with or without additional clinical/biochemical parameters but none were proved to be perfect [6, 7, 12, 13, 17, 18]. Importantly, no single histopathological parameter is pathognomonic of ACC. In the latest clinical guidelines co-authored by the European Society of Endocrinology (ESE) and the European Network for the Study of Adrenal Tumors (ENS@T), the Weiss scoring system is recommended as the preferred system for classifying benign and malignant adrenocortical tumors and is the most widely employed system [2, 19]. It was first introduced in 1984 and comprises nine histological criteria (Table 3), which are graded according to the stringent morphological assessment of tumor structure, cytological features, and invasive tumor properties under light microscopy [2, 8, 20]. The presence of three or more of which was reported to be highly correlated with subsequent malignant behavior and is diagnostic of ACC [10]. The reported sensitivity and specificity were about 86–95% and 90%, respectively in expert hands [14]. However, borderline cases of adrenocortical neoplasm with uncertain malignant potential may challenge the reliability of the Weiss system, and these tumors often have a Weiss score of 1–3 [2, 8, 11]. It has been observed that adrenocortical tumors with a Weiss score of 3 or less could eventually turn out to be malignant ACC, posing particular challenges in the postoperative surveillance in these patients [3, 8, 11, 21]. Interobserver reproducibility is another factor affecting the accuracy of the Weiss scoring system as some features, namely diffuse growth, necrosis, sinusoidal, vascular and capsular invasion, and nuclear atypia, are determined subjectively [3, 8, 22]. Other factors potentially undermining the diagnostic performance of the Weiss scoring system include the inadequacy of tumor sampling, increasing prevalence of incidentally detected small ACC, low applicability among nonexpert pathologists, and ACC variants [8, 11].

The imperfection of the Weiss scoring system has instigated the development of a more unequivocal pathological classification system over the past three decades. In 2002, Aubert et al. modified the Weiss scoring system to include only five of the original nine items with a maximum score of 7. Being easier to use in clinical practice and more reproducible, the modified Weiss score has a reported specificity of 96.9% (vs. 90.2% in the original score) [7]. More recently, the reticulin algorithm and the Helsinki score (Table 3) have been validated and are shown to be highly accurate in differentiating between benign and malignant adrenocortical tumors with 100% or nearly 100% sensitivity and specificity [3, 4, 14]. The rationale of the reticulin algorithm relies on the histochemical assessment of the reticulin network, which is commonly disrupted in ACC [12]. On the other hand, the Helsinki score, developed by the University of Helsinki, is based on a stepwise regression analysis of the nine criteria in the Weiss scoring system and the proliferation index Ki-67 in 177 consecutive adult patients with primary adrenocortical tumors [13]. The final model consists of 3 criteria only and correlates with prognosis [23]. While interobserver agreement and applicability among pathologists remain a drawback for both of these systems, these could be overcome by training and are endorsed by the ESE/ENS@T guidelines for the assessment of tumors of borderline malignant potential [2, 14, 24]. However, these systems were either not applicable (reticulin algorithm) or not available (Helsinki score) at the time when our patients received their first operation. Latest effort and research have been put into the development of novel immunohistochemical (e.g., insulin-like growth factor-2 [IGF-2], MAD2L1, CNNB1, and markers for cell proliferation, mitotic spindle regulation [p53, BUB1Bm HURP, and NEK2], and DNA damage repair [PBK and y-H2Ax]) and genomic markers (e.g., aberrations in methylome and transcriptome, micro-RNA expression, chromosomal aberrations, and DNA mutation profile) that can improve the distinction between ACA and ACC [14]. Although these might be valuable in diagnosing ACC, especially when the diagnosis cannot be made definitively on histopathological and/or clinical grounds, protocol standardization, availability, cost, and large genetic diversity of ACC are the main hindrances of their routine incorporation in clinical practice.

The diagnostic difficulty of ACC is well exemplified in the present two cases where the features of overt malignancy were not evident pathologically. The presence of only a diffuse growth pattern and <25% clear cells of the tumor volume in Patient A, and the presence of borderline mitosis of 5/50 HPF and regressive changes (fibrosis and hemorrhage) in Patient B would not be sufficient to classify either tumor as ACC under the Weiss scoring system or other systems (Tables 4 and 5). Only the Van Slooten system would classify Patient B's tumor as ACC retrospectively (Table 5). Laparoscopic surgery may have further complicated the diagnostic challenge during which the tumor was fragmented upon surgical manipulation, thus affecting the integrity of the tissue specimens to be examined and making a detailed assessment of capsular and stromal invasion difficult. Laparoscopic adrenalectomy for ACC has been linked to inferior survival and higher recurrence rates, where rupture of the tumor capsule and peritoneal seeding are recognized as major factors for recurrence [9, 19]. Nevertheless, the identification of the above histopathological features, either individually or in combination, should alert the clinician of the malignant potential of the tumor. Furthermore, the presence of focal myxoid change, which is not incorporated in any of the abovementioned systems, has been reported to be associated with ACC having a low Weiss score [3, 8, 25].

Apart from the above histopathological clues that speak for the potential malignant behavior of an adrenocortical tumor, several clinical, radiological, and biochemical features in our present cases should have been recognized as incongruent with a simple benign ACA in retrospect. These included the large size of the tumor on presentation, an imaging phenotype inconsistent with adenoma, and co-secretion of adrenal cortisol and androgens and their precursors. The presence of both having a diameter greater than 4 cm and a CT tumor attenuation >10 HU are shown to be highly sensitive to adrenal malignancy [26–28]. A more recent prospective study published in 2020 by Bancos et al. suggested that a tumor diameter of 4 cm and unenhanced CT tumor attenuation of 20 HU may be more appropriate threshold values for the consideration of an ACC. In their study of 2169 participants from 14 specialist centers in 11 countries involving 98 (4.9%) patients with ACC, increasing the unenhanced CT tumor attenuation threshold from the recommended 10HU to 20 HU (and keeping a diameter threshold of 4 cm) increased the specificity for the detection of ACC (64% vs. 80·0%) while maintaining sensitivity (100% vs. 99%) in patients with newly identified adrenal masses more than 1 cm after exclusion of phaeochromocytoma and patients with a history of cancer [29]. Other radiological features suggestive of ACC include inhomogeneous appearance, presence of calcification, central low attenuation with a thin enhancing rim (representing tumor necrosis), and contrast washout characteristics concordant with non-adenoma (i.e., absolute washout <60% and relative washout <40%) [1, 30–32]. The latest ESE guideline on adrenal incidentaloma stated that further washout CT characterization for inhomogeneous lesions should not be performed and recommended surgical resection in unilateral adrenal masses with radiological suspicion of malignancy, recognizing the potential of ACC in these lesions [27].

The pattern of steroid secretion may also indicate if an adrenal lesion is an ACC as disorganized steroidogenesis is almost always observed in ACC. Co-secretion of androgens and cortisol, secretion of immature steroid precursors, or secretion of estradiol in males are highly suspicious for ACC [14]. In recent decades, USP has emerged as an important diagnostic and surveillance tool in the evaluation of adrenal tumors. Specifically, USP can reveal the specific fingerprints of ACC coinciding with immature and disorganized early stage steroidogenesis and has been shown to differentiate malignant from benign adrenocortical tumors with high sensitivity and specificity [33, 34]. The adrenal steroid precursor, tetrahydro-11-deoxycortisol (THS), a 11-deoxycortisol metabolite, was found to be the most discriminative steroid in differentiating ACC from ACAs in a study supported by ENS@T [33]. Another Dutch study also showed that urinary THS excretion at a cut-off value of 2.35umol/24 hr differentiated ACC from other adrenal disorders with a sensitivity of 100% and specificity of 99% [35]. Of note, USP can detect significantly increased steroid precursor excretion in those ACCs that would have been classified as endocrinologically inactive based on routine endocrine workup and can serve as a surveillance marker. Similarly, urine steroid metabolomics has lately been shown to improve the diagnostic accuracy of ACC in patients with newly found adrenal masses in addition to CT tumor attenuation values and tumor diameter by virtue of the specific biological secretory signature of ACC [29]. Apart from USP, different groups have started to explore the use of plasma steroid metabolome profiling by LS-MS/MS in discriminating between ACA and ACC. Their findings found an increase in 11-deoxycortisol, 11-deoxycorticosterone, androstenedione, and 17-OHP levels in patients with ACC compared to those with ACA and suggested that plasma steroid profiling may emerge as a potential diagnostic tool for ACC [36, 37]. However, as the method is not widely available at present and its diagnostic value on top of USP is still unknown, further evaluation should be performed before its routine clinical use.

In Hong Kong, USP using gas chromatography-mass spectrometry (GC-MS) identified 3*α*, 16*α*, 20*α*-pregnenetriol and 3*β*, 16*α*, 20*α*-pregnenetriol to be highly specific for ACCs in addition to THS and these steroid metabolites were also observed in Patient A. USP can identify residual or recurrent ACCs even before the disease becomes radiologically apparent and is useful in the surveillance and monitoring of recurrent disease [34, 38]. Chortis et al. suggested that the appearance of abnormal metabolites may predate imaging abnormality by 2 months based on their analysis of 135 patients with ACCs after R0 resection. They also proposed that the availability of preoperative urine samples considerably improved the ability of USP to detect ACC recurrence [39].

In terms of treatment, complete surgical removal is the only curative therapy for localized ACC. In patients with unresectable tumors or incomplete tumor resection, the prognosis is significantly worse [1, 9, 40]. However, despite complete surgical removal, the recurrence rate can be as high as 50–80% [40, 41]. Predictors for recurrence include advanced disease stage, incomplete surgical resection, hypercortisolism, laparoscopic adrenalectomy, and high proliferation rate (Ki-67 proliferation index) [9, 41]. Molecular markers such as somatic gene mutations (*TP53, ZNFR3, CTNNB1, PRKAR1A, CCNE1, TERF2, TERT, CDK*s, and genes involved in histone modification [*MLL, MLL2, MLL4*] and chromatin remodeling [*ATRX, DAXX*]), alterations in methylome and transcriptome, miRNAome, and chromosomal aberrations are emerging tools for further prognostication of ACC but yet to be incorporated into clinical practice due to the resource-intensive nature of pan-genomic bioinformatic analysis [14, 40–44]. The use of adjuvant mitotane is controversial as there is much uncertainty regarding its efficacy in preventing recurrence and improving mortality. A meta-analysis in 2018 including 5 retrospective studies and 1249 patients found that adjuvant mitotane was associated with longer recurrence-free survival (HR 0.62; 95% CI 0.42–0.94) and overall survival (HR 0.69; 95% CI 0.55–0.88). In another meta-analysis by ESE and ENS@T in 2018, favorable effects were also observed on recurrence (HR 0.7; 95% CI 0.5–1.1) and mortality 0.7 (95% CI 0.5–0.9) in patients receiving adjuvant mitotane, while acknowledging the fact that all six studies included in the meta-analysis were non-randomized with the potential of a confounding effect [2, 41]. Despite the absence of completely convincing evidence, the ESE and ENS@T guidelines recommended that adjuvant mitotane should be offered to patients at high risk of recurrence (Stage III or IV, R1 resection, or Ki67 > 10%), and the duration of treatment should last for at least 2 years but not longer than 5 years. For patients at low to intermediate risk of recurrence (Stage I/II, R0 resection, and Ki-67 </ = 10%), adjuvant mitotane should be individualized, while for patients with adrenocortical tumors of uncertain malignant potential, adjuvant therapy should not be started [2, 24].

For patients who develop recurrent disease at least 12 months beyond the initial surgery, complete surgical removal of the recurrence is still recommended if it is achievable [1, 2, 19, 45]. At the same time, mitotane should be commenced as soon as possible if it has not been given before as in our patients, while acknowledging the fact that the objective response rate to mitotane in metastatic ACC is at best 24% [2, 9, 45]. In our patients, there seemed to be initial stabilization of tumor growth for the first few months after mitotane in Patient B, while a response was not observed in Patient A. In selected patients (e.g., patients with severe hormone excess), debulking surgery may be offered as palliation if >80% of the tumor can be removed, the hormonal excess remains uncontrolled despite maximally tolerated medical therapy, and the patient is deemed a surgical candidate [2, 45]. In line with the recommendation, our first patient did enjoy an extended period of survival with good quality of life after palliative surgery. Further localized therapy and systemic therapy should be individualized and discussed in a multidisciplinary team. In patients with progressive metastatic ACC not amenable to surgery or mitotane monotherapy, systemic chemotherapy with EDP combined with mitotane represents the current standard of care as supported by the FIRM-ACT trial, the largest randomized controlled trial in ACC to date. However, the response rate remained dismal with only a modest improvement in progression-free survival of 5 vs. 2.1 months (HR 0.55, *p* < 0.001) and no effect on overall survival as compared to streptozocin plus mitotane (14.8 vs. 12 months, HR 0.79, *p*=0.07) [46]. EP combined with mitotane might have modest activity against ACC with an objective response rate of 11% [47]. This regime was employed as the initial palliative chemotherapy in Patient A after balancing her fair performance status at that juncture and risk of toxicity with EDP. The combination of gemcitabine and capecitabine has been proposed as second- or third-line therapy in heavily pretreated patients with metastatic ACC and has been reported to achieve a 4 months' non-progression rate of 46%. The lack of a control arm is a major limitation of the study [48]. Other systemic therapies involving IGF2/IGF-I receptor inhibition (linsitinib, cixutumumab), mTOR inhibition (everolimus, sirolimus, temsirolimus), selective or multi-tyrosine kinase inhibition including EGFR, VEGF/VEGFR2, RET, and FGFR inhibition (gefinitib, erlotinib, sorafenib, sunitinib, lenvatinib, dovitinib, axitinib, nilotinib, cabozantinib), sterol O-acyltransferase-1 (SOAT) inhibition (nevanimibe) and immunotherapy (avelumab), either alone and in combination, were ineffective in achieving clinically meaningful outcomes [19, 40, 49, 50]. A recent phase II study with pembrolizumab may hold a sign of promise for immune checkpoint inhibitor for advanced ACC, in which an objective response rate of 23% and *s* disease control rate of 23% were observed in 39 patients over a median follow-up of 17.8 months [51]. Other potential emerging therapies or therapeutic targets under investigation include peptide receptor radionuclide therapy (PRRT), cyclin-dependent kinase (CDK) inhibition, Notch inhibition, Ras/Raf/MARK/ERK pathway inhibition, murine double minute (MDM2) inhibition, and Poly-ADP-ribose polymerase (PARP) inhibition [45, 50, 52].

## 4. Conclusion

Adrenocortical carcinoma is an aggressive endocrine cancer in which therapeutic options are often limited and prognosis becomes gloomy once metastatic disease is manifested. It is also a heterogeneous disease with varying clinical behavior, where a small subset of patients harboring low-grade well-differentiated tumors may demonstrate a more indolent clinical course [52]. It should not be overstated that histology can be deceptive in these patients and clinicians should be aware of the potential limitation of and overreliance on the Weiss scoring system in the differentiation of benign and malignant adrenocortical tumors. Other clinical parameters, particularly the pattern of hormonal secretion, urinary steroid profiling, and imaging features, should be incorporated into the prediction of the malignant potential of these “borderline” tumors, thus assisting the subsequent surveillance strategies. Mitotane therapy forms the cornerstone of treatment in metastatic ACC, and in combination with etoposide, doxorubicin, and cisplatin represents the currently best evidence-based treatment in patients with advanced ACC.

## Figures and Tables

**Table 1:** Biochemical investigations of Patient A in 2012 and 2016.

Test	Date			Reference Range
	8/2012		5/2016	
Serum				
Renin	0.45		2.91	0.15–2.33 ng/mL/hr
Aldosterone	267		<80	28–444 pmol/L
Estradiol	83		222	<147 pmol/L
Testosterone	3.8		4.2	0.28–1.2 nmol/L
DHEA-S	29.1		33.0	0.5–5.6 umol/L
1 mg overnight dexamethasone suppression test (Cortisol)	518		666	<50 nmol/L
ACTH	11		<5	10- 46 pg/ml
Urine				
24-h urinary free cortisol	188		2798	24–140 nmol/ day
24-hr urine adrenaline	26		—	<110 nmol/day
24-hr urine noradrenaline	181		—	<440 nmol/day
24-hr urine metanephrine	105		—	<275 nmol/day
24-hr urine normetanephrine	120		—	<240 nmol/day
Urinary steroid profiling	Markedly increased free cortisol, cortisol metabolites, and androgen. Also, elevated levels of THS, 3-*α*, 16-*α*, 20-*α*-pregnenetriol, 3-*β*,16-*α*, 20-*α-*pregnenetriol, and 17-*β* androstenediol		—	
Low dose dexamethasone suppression test				
	Day 0	Day 2		
Cortisol	469	450	—	<50

**Table 2:** Biochemical investigations of Patient B in 2011 and 2016.

Test	Date						Reference Range
	11/2011			12/2016			
Serum							
Renin	0.75			—			0.15–2.33 ng/mL/hr
Aldosterone	81			—			28–444 pmol/L
Estradiol	—			<133			<147 pmol/L
Testosterone	—			0.6			0.28–1.2 nmol/L
DHEA-S	—			33.0			0.5–5.6 umol/L
1 mg overnight dexamethasone suppression test (Cortisol)	612			666			<50 nmol/L
ACTH	13			<5			10–46 pg/ml
Urine							
24 h	722			689			24–140 nmol/ day
24-hr urine adrenaline	32			—			<110 nmol/day
24-hr urine noradrenaline	298			—			<440 nmol/day
24-hr urine metanephrine	36			—			<275 nmol/day
24-hr urine normetanephrine	106			—			<240 nmol/day
Urinary steroid profiling	Free cortisol and cortisol intermediates and metabolites were grossly elevated			Moderate to large increases in free cortisol and cortisol metabolites. 17-OHP metabolites were grossly elevated			
Low-dose dexamethasone suppression test							
	Day 0	Day 2		Day 0	Day 2		
Cortisol	632		558		348	338	<50

**Table 3:** Summary of published classification systems for adrenocortical tumors and their diagnostic threshold for adrenocortical carcinoma.

Criteria	Weiss [6]	Hough [16]	Van Slooten [17]	Modified Weiss [7]	Reticulin algorithm [12]	Helsinki score [13]
Nuclear atypia (Fuhrman grade III or IV)	1	0.39	2.1			
Nuclear hyperchromatism			2.6			
Abnormal nucleoli			4.1			
Atypical mitoses	1			1		
Mitoses > 5/50 HPF >10/100 HPF > 2/10 HPF	1	0.60	9.0	2	Optional	3
Clear cells < 25% of total tumor volume	1			2		
Architecture diffuse loss of normal structure	1	0.92	1.6			
Venous invasion	1	0.92				
Capsular invasion	1	0.37		1		
Sinusoidal invasion	1					
Vascular invasion					Optional	
Capsular or vascular invasion	1		3.3			
Necrosis	1	0.69		1	Optional	5
Regressive changes (necrosis, hemorrhage, fibrosis, or calcification)			5.7			
Thick fibrous band		1.00				
Response to ACTH (17-hydroxysteroids increased two times after 50 mcg of IV ACTH)		0.42				
Urinary 17-ketosteroids (10 mg/1 g creatinine 24 h)		0.50				
Cushing's syndrome with virilism, virilism alone, or no clinical manifestations		0.42				
Weight loss (>10 Ib/3 months)		2.00				
Tumor mass (>100 g)		0.60				
Disruption of reticulin network					Mandatory	
Proliferative index (Ki-67) in the most proliferative area of tumor						1-100
Diagnostic threshold of ACC	≥3	>2	≥8	≥3	Mandatory criteria + any 1 optional	3 × mitoses + 5 necrosis + PI in % by Ki-67 > 8.5

**Table 4:** Suggested pathological diagnosis of the adrenocortical tumor at the first operation of Patient A based on different classification systems on retrospective pathology review. The tumor would have been classified as ACA by the majority of systems.

Clinical features	Weiss	Hough	Van Slooten	Modified Weiss	Reticulin algorithm	Helsinki score
Case 1	Diffuse growth clear cells < 25%	Diffuse growth (0.92). Cushing's syndrome with virilism (0.42). Insufficient data on ACTH response, 24-hr urinary 17-ketosteroids, and tumor mass	Loss of normal architecture (1.6)	Clear cells <25% (2)	Mandatory criteria not available but optional criteria not met	Criteria on mitoses and necrosis not met and PI not available
Score	2	At least 1.34	1.6	2	—	—
Classification based on system	ACA	Not clear	ACA	ACA	ACA	Not clear

**Table 5:** Suggested pathological diagnosis of the adrenocortical tumor at the first operation of Patient B based on different classification systems on retrospective pathology review. Only the Van Slooten system would have classified the tumor as ACC unequivocally.

Clinical features	Weiss	Hough	Van Slooten	Modified Weiss	Reticulin algorithm	Helsinki score
Case 2	No matching criteria	Thick fibrous band (1.00). Insufficient data on ACTH response, 24-hr urinary 17-ketosteroids, and tumor mass	Nuclear hyperchromatism (2.6). Regressive changes (5.7)	No matching criteria	Mandatory criteria not available and optional criteria not met	Criteria on mitoses and necrosis not met and PI not available
Score	0	At least 1.00	8.3	0	—	—
Classification based on system	ACA	Not clear	ACC	ACA	ACA	Not clear

## Data Availability

All data generated or analyzed during this study are included in this published article.

## Abbreviations

17-OHP: 17-hydroxyprogesterone
ACA: Adrenocortical adenoma
ACC: Adrenocortical carcinoma
CDK: Cyclin-dependent kinase
CT: Computed tomography
EDP: Chemotherapy regime containing a combination of etoposide, doxorubicin, and cisplatin
EGFR: Epidermal growth factor receptor
ENS@T: European Network for the Study of Adrenal Tumors
EP: Chemotherapy regime containing a combination of etoposide and cisplatin
ESE: European Society of Endocrinology
^18^FDG-PET-CT: ^18^F-fluorodeoxyglucose positron emission tomography/ computed tomography FGFR:
FIRM-ACT: First International Randomized Trial in Locally Advanced and Metastatic Adrenocortical Carcinoma Treatment
GC-MS: Gas chromatography-mass spectrometry
HPF: High power field
HU: Hounsfield unit
IGF-1: Insulin-like growth factor 1
IGF-2: Insulin-like growth factor 2
MDM2: Mouse double minute 2 homolog
mTOR: Mammalian target of rapamycin
PARP: Poly-adenosine diphosphate-ribose polymerase
PD-L1: Programmed-death ligand 1
PRRT: Peptide receptor radionuclide therapy
RET: Rearranged during transfection
SOAT: Sterol O-acyltransferase-1
SUVmax: Maximum standardized uptake value
THS: Tetrahydro-11-deoxycortisol
US: Ultrasound
USP: Urinary steroid profiling
VEGF1: Vascular endothelial growth factor 1
VEGFR2: Vascular endothelial growth factor receptor 2.
